# Advances in 3D skin bioprinting for wound healing and disease modeling

**DOI:** 10.1093/rb/rbac105

**Published:** 2022-12-19

**Authors:** Mengde Zhang, Chao Zhang, Zhao Li, Xiaobing Fu, Sha Huang

**Affiliations:** Research Center for Tissue Repair and Regeneration affiliated to the Medical Innovation Research Department, PLA General Hospital and PLA Medical College, 28 Fu Xing Road, Beijing 100853, China; Research Center for Tissue Repair and Regeneration affiliated to the Medical Innovation Research Department, PLA General Hospital and PLA Medical College, 28 Fu Xing Road, Beijing 100853, China; School of Medicine, Nankai University, 94 Wei Jing Road, Tianjin 300071, China; Research Center for Tissue Repair and Regeneration affiliated to the Medical Innovation Research Department, PLA General Hospital and PLA Medical College, 28 Fu Xing Road, Beijing 100853, China; Research Center for Tissue Repair and Regeneration affiliated to the Medical Innovation Research Department, PLA General Hospital and PLA Medical College, 28 Fu Xing Road, Beijing 100853, China; School of Medicine, Nankai University, 94 Wei Jing Road, Tianjin 300071, China; Research Center for Tissue Repair and Regeneration affiliated to the Medical Innovation Research Department, PLA General Hospital and PLA Medical College, 28 Fu Xing Road, Beijing 100853, China

**Keywords:** 3D bioprinting, skin, stem cells, regeneration

## Abstract

Even with many advances in design strategies over the past three decades, an enormous gap remains between existing tissue engineering skin and natural skin. Currently available *in vitro* skin models still cannot replicate the three-dimensionality and heterogeneity of the dermal microenvironment sufficiently to recapitulate many of the known characteristics of skin disorder or disease *in vivo*. Three-dimensional (3D) bioprinting enables precise control over multiple compositions, spatial distributions and architectural complexity, therefore offering hope for filling the gap of structure and function between natural and artificial skin. Our understanding of wound healing process and skin disease would thus be boosted by the development of *in vitro* models that could more completely capture the heterogeneous features of skin biology. Here, we provide an overview of recent advances in 3D skin bioprinting, as well as design concepts of cells and bioinks suitable for the bioprinting process. We focus on the applications of this technology for engineering physiological or pathological skin model, focusing more specifically on the function of skin appendages and vasculature. We conclude with current challenges and the technical perspective for further development of 3D skin bioprinting.

## Graphical Abstract

**Figure:**
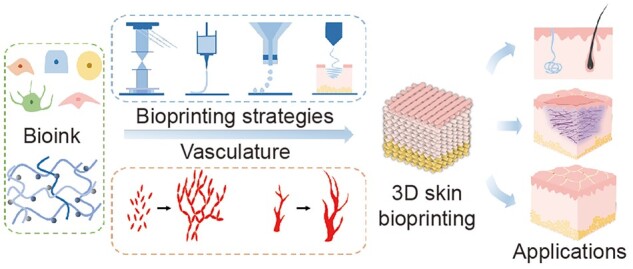

## Introduction

Human skin is a complex three-layered structure formed by the combined, functional organization of multiple cell types. The cells in these layers are highly specialized and gathered to perform distinctive functions [1]. Various skin-related injuries are drastically increasing due to traumatic damage and disease [2]. Currently, the clinical treatments for the repair or replacement of missing or malfunctioning human skin are limited by the availability of healthy donor tissue and immune rejection of donated tissue [3, 4]. In the search for alternatives to conventional treatment strategies for skin replacement and repair, tissue engineering approaches are being explored as a promising solution.

In the past three decades, skin tissue engineering has shown great promise in the application of wound healing strategies and tissue regeneration. In some practice, such as deep burns and wounds, artificial skin substitutes are upcoming alternatives to traditional treatment. Recent advancements in stem cell biology and various innovative biomaterials have further provided a tremendous springboard for researchers in developing and manipulating tissue-engineered skin for improved skin regeneration and wound healing. However, low adherence to the wound bed, the inability to reproduce skin appendages and inadequate vascularization limits their utilization for restoration of normal skin anatomy on a routine basis [5, 6].

Three-dimensional (3D) bioprinting is a cutting-edge technology to fabricate precisely controlled architecture with highly reproducibility and repeatability through a layer-by-layer building process [7, 8]. Therefore, in the skin tissue engineering field, it can provide an excellent alternative for biomimetic scaffold fabrication by accurately positioning multiple cell types as well as biochemical and biophysical cues simultaneously into complex multi-layer architectures that better represent the structural and functional complexity of skin.

**Figure 1.**
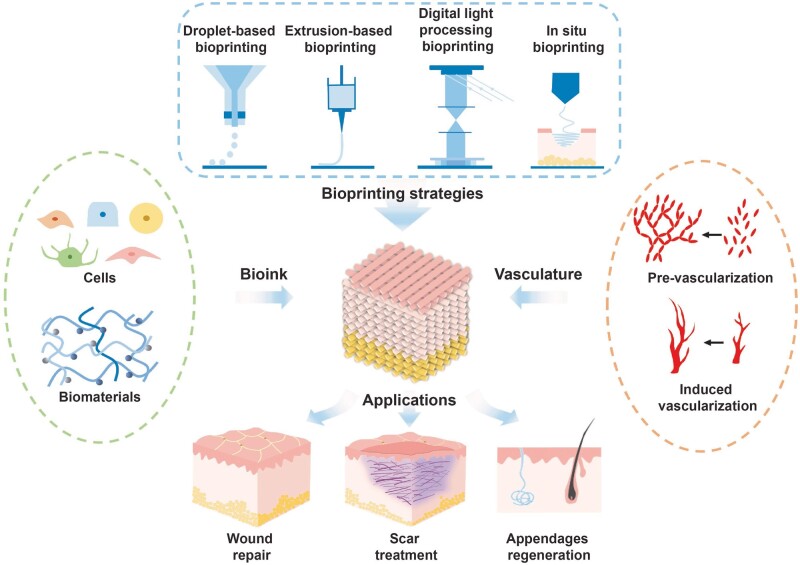
Schematic diagram of essential elements pertaining to the application of skin bioprinting.

Although still in its infancy considering the complexity and functionality, 3D bioprinting approaches have been widely studied for skin tissue engineering and regeneration, both in research and clinical applications [9–11]. When engineering a 3D skin models *in vitro*, the requirements of cells, bioink and bioprinting process must be considered in a biomimetic and spatiotemporal manner. In this manner, 3D structural characteristics and physical properties can substantially enhance the biological performance and function through appropriate cell–cell and cell–extracellular matrix (ECM) communication. Therefore, in this review, we focus on general principles of cell and bioink choice or prepare techniques, and other essential elements pertaining to the application of 3D bioprinting technologies for generating physiological or pathological skin model. We propose a strategy of bioprinting the representative microenvironmental characteristics of native skin and further regenerating skin with appendages. Furthermore, we present recent advances in 3D skin with vascular network integration and discuss current challenges and exciting opportunities of 3D skin bioprinting toward recreating the complex, heterogeneous architecture of functional skin that further fundamental research and translational medicine (Fig. 1).

## Bioinks for skin bioprinting

Bioinks play a critical role in 3D bioprinting to recapitulate the physiological and pathological environment. They are designed elaborately with a hydrogel form of biomaterials loaded with specific cell types [12]. Yet, creating an equivalent that faithfully mimics the intricate composition, structure and function of skin is still a challenge [13]. This puts forward high demands on the selection of both biomaterials and cells for bioinks. In this section, suitable biomaterials and desired properties are briefly summarized at the beginning. Then different cells used for skin bioprinting are reviewed.

### Biomaterials

#### Category

Natural polymers and synthetic polymers are the common biomaterials used for 3D bioprinting [14]. Many natural polymers existed in natural ECM or extracted from marine organisms and natural substances have been widely used in skin bioprinting such as gelatin, collagen, alginate, chitosan and fibrin [15]. They mostly have a high water content, which is similar to the native ECM. Excellent biocompatibility, degradability and low immunogenicity make them preferable in the applications of skin bioprinting. However, the poor mechanical properties and potential immunogenic reactions restrict their applications [11]. Synthetic polymers can be generally classified into nonbiodegradable and biodegradable polymers [16]. Nonbiodegradable synthetic polymers, among which polyethylene glycol (PEG) is the most widely exploited, are more frequently used for engineering bone and cartilage instead of engineering skin [16]. Biodegradable synthetic polymers, such as poly(lactic acid) and poly(ε-caprolactone) (PCL), can degrade naturally at a certain rate [17]. Controllable mechanical properties and structure stability are the outstanding advantages of synthetic polymers [18]. In practical applications, synthetic materials are often modified and blended with natural materials to enhance their properties. Table 1 provides a brief summary of the characteristics, disadvantages and applications of several common natural polymers.

**Table 1.** Characteristics and applications of common polymers in skin bioprinting

Category	Polymers	Gelation mechanism	Biodegradability	Cell binding domains	Disadvantages	Applications				
						Common usage	Target structure	Cells	Bioprinting strategy	Ref.
Natural polymers	Gelatin	Thermal Chemical	Yes	Yes	Low viscosity Mechanical instability	GelMA Alginate/Gelatin	Dermis Epidermis/dermis	Fbs AECs/MSCs	Extrusion Extrusion	[19] [20]
	Collagen	Thermal	Yes	Yes	Low viscosity Mechanical instability Slow gelation rate Fast degradation rate	Collagen Collagen Collagen/GelMA	Epidermis/Dermis Epidermis/dermis Epidermis/dermis	KCs/Fbs/MCs KCs/Fbs KCs/Fbs/MCs	Droplet Droplet Extrusion	[21] [22] [23]
	Alginate	Ionic	No	No	Low biodegradability Lack cell adhesion site	Alginate/Gelatin Alginate/PLGA Alginate/NFC/CMC	Sweat gland Epidermis/dermis Dermis	EPs KCs/Fbs Fbs	Extrusion Extrusion Extrusion	[24] [25] [26]
	Chitosan	pH	Yes	Yes	Mechanical instability Poor solubility Slow gelation rate	Chitosan Chitosan/gelatin	Epidermis/dermis Dermis	KCs/Fbs Fbs	Extrusion Extrusion	[27] [28]
	Fibrin	Thrombin	Yes	Yes	Mechanical instability High viscosity Low shear thinning	Fibrin	Epidermis/dermis	KCs/Fbs	Extrusion	[29]
Synthetic polymers	PEG	Thermoplastic	NO	NO	Hydrophobicity Long degradation time Lack cell adhesion site	PEG/chitosan/genipin Silk fibroin/PEG	Epidermis/dermis Epidermis/dermis	KCs/Fbs KCs/Fbs	Extrusion Droplet	[30] [31]
	PLA	Thermoplastic	Yes	No	Hydrophobicity Long degradation time Lack cell adhesion site	PLA/chitosan/HA	Dermis	Fbs	Extrusion	[32]
	PCL	Thermoplastic	Yes	No	Hydrophobicity Long degradation time Lack cell adhesion site	PCL/collagen	Epidermis/dermis	KCs/Fbs	Inkjet	[33]

AECs, amniotic epithelial cells; CMC, carboxymethyl cellulose; Eps, epidermal progenitors; Fbs, fibroblasts; GelMA, gelatin methacryloyl; HA, hyaluronic acid; KCs, keratinocytes; MCs, melanocytes; MSCs, mesenchymal stem cells; NFC, nano-fibrillated cellulose; PCL, poly(ε-caprolactone); PEG, polyethylene glycol; PLA, poly(lactic acid); PLGA, poly(lactic-co-glycolic acid).

In addition, the highly dynamic wound healing process and the complex microenvironment of skin diseases make higher requirements on biomaterials [34, 35]. Bioinks based on decellularized extracellular matrix (dECM), which can retain part of the structural and functional properties of natural ECM, have been considered a promising choice in recent years [36]. Since dECM is obtained from heterogeneous natural tissues, its greatest advantage distinguished from other biomaterials is that it can provide a greater diversity of structural, chemical and biological cues [37, 38]. It also shows good biocompatibility and printability [39]. Several bioinks based on dECM perform well in the construction of skin substitutes [10, 40–42]. However, removal of histocompatibility complexes from native tissues by physical, chemical or biological means is a necessary step to avoid immune and rejection reactions after transplantation, which inevitably leads to the destruction of the microstructure and bioactive substances in ECM [43]. Optimization of the decellularization process is still a challenge to be overcome in the future.

#### Physical and chemical properties

Biomaterials not only act as temporary ECM scaffolds, affecting the biological behavior of cells, but also determine whether printing can be carried out successfully [44]. Ideality, during the printing process, bioinks should have strong plasticity and ensure cell viability. Upon completion of printing, it is important to maintain structural stability for a certain period of time and regulate the behavior of cells of the printed construction. When the printed tissue is transplanted on wound or defect of organism, it is necessary to ensure the interaction and integration between them [45]. In view of the above, biomaterials for skin bioprinting should be evaluated according to the following properties: (i) biocompatibility and degradability; (ii) printability; and (iii) biomechanical and biochemical properties [46].

First and foremost, the biomaterials used for bioink should be hypotoxic to encapsulated cells and implanted host as well as their degradation products [44]. In addition to direct toxic damage, negative immune responses should also be avoided. Immune rejection is a great challenge for the *in vivo* application of skin substitutes [44]. As for the skin wound treatment, biomaterials with excellent biocompatibility could avoid excessive inflammatory reactions and infections. In this term, using biomaterials with bio-inertia or adding bioactive substances to bioinks may be effective [47]. Immunomodulatory biomaterials, which can induce positive immune responses to accelerate wound healing on their own, have also gained attention in recent years. Griffin *et al*. described a scaffold containing microporous annealed particle for wound treatment. It showed the adaptive immune response from this scaffold induced cutaneous regenerative healing [48]. Meanwhile, biomaterials should have adhesive sites with cells for their adhesion, proliferation and differentiation. Alginate is a commonly used natural polymer due to its good biocompatibility and printability [49]. It can be easily cross-linked into hydrogels by divalent cations, but it needs to be used with other biomaterials since the lack of adhesive sites with cells. De Santis *et al*. described a tissue-specific hybrid bioink composed of alginate and dECM. The presence of dECM provided adhesive sites for cells and significantly increased the viability and proliferation of epithelial and endothelial cells (ECs). *In vivo*, 3D-bioprinted constructs promoted angiogenesis and minimized the foreign body response [50]. On the other hand, degradability is the key to accelerate wound repair after implantation by cell delivery and intercellular communication [44]. From this aspect, biomaterials with tunable degradation rates similar to natural ECM are preferred. Alginate/gelatin bioink, performing excellent printability and structural fidelity, is widely used in skin bioprinting [51]. However, the absence of enzymes in mammals that can break down alginate scaffold causes alginate residues in the body, limiting the biological behavior of cells [52]. Huang *et al*. developed a degradable alginate/gelatin bioink by adding alginate lyase and coordinated the degradation rate and skin wound healing. Through this approach, 3D-bioprinted constructs demonstrated better degradability both *in vitro* and *in vivo*. Cellular behaviors, such as cell adhesion, extension and proliferation were also promoted [52].

Printability ensures that the bioink forms a stable model consistent with the predetermined structure without damaging the cells [53]. Although the density and size of cells in bioinks might affect the printability [13], the biomaterials primarily determine the printability. Printability of bioink is defined by the rheological properties of its main biomaterials in hydrogel phase [53]. Bioinks need to be injected smoothly under certain pressure in the printing process and maintain stable structure after printing [54]. Viscosity is one of the basic parameters to evaluate the rheological properties of biomaterials, it refers to the resistance of a fluid to flow upon application of stress [55, 56]. While higher viscosity means stiffer bioink, it may cause damage to cells. Lower viscosity is more friendly to cells, but makes it difficult to form stable structures [13]. Balancing a proper viscosity is an important aspect for the preparation of bioinks. Another important property of biomaterials is shear thinning. Under the high shear rate inside nozzle during printing, shear thinning causes a decrease in viscosity to avoid clogging, as well as avoid damaging cells by high shear stress [57]. The reduction of shear rate after printing in turn increases the viscosity of bioinks, thus ensuring the fidelity of the printed structure. Many approaches have been used to adjust rheological properties and enhance the printability of bioinks, among which changing the concentration or ratio of different biomaterials is more popular [58]. Huang *et al*. found that the printability of bioink can also been influenced by the solvent through tuning the ionic strength. By adjusting formulations to customize bioink with different solvent ionic strength, the printing performance of gelatin–alginate bioink and the behavior of the embedded stem cells was optimized [59].

The biomaterials used in bioinks are not simply provide scaffolds, they also deliver biomechanical and biological signals for cell survival, which are essential for the function of skin [9]. The distribution and density of ECM proteins in the skin varies across regions, stages of wound healing and pathological states [60], which leads to differences in the biomechanical properties of the skin. Recent studies have declared that mechanosensitive proteins inside cells can sense biomechanical changes of the environment, which influence long-term cellular processes such as differentiation and fibrosis through continuous sensing and cellular memory [61]. Meanwhile, the behavior of cells also generates mechanical forces and deformations in biological materials, which in turn influence the surrounding cells and microenvironment. Therefore, the category of biomaterials should be appropriately selected and mixed according to the required biomechanical properties when preparing different skin substitutes. There are many approaches to optimize the biomechanical properties of bioink, such as utilizing functionalized polymers, supramolecular hydrogels and surface modifications [58]. However, these methods are more complex and costly. Huang *et al*. explored an easy-to-use method of mechanical enhancement by adding bioactive glass nanoparticles to gelatin–alginate bioink. The mechanical strength, printability and cellular behavior were improved in the bioprinted constructs [62, 63]. Although many physical cues, such as geometry, porosity and topology [64], have been shown to affect various aspects of cell behavior, viscoelasticity is an important factor of mechanical properties for biomaterials to be considered in recent researches. Viscoelasticity is a near-universal feature of living tissues and ECMs [61]. The viscoelastic natural skin exhibits both elastic solid properties that restore the original structure and viscous liquid characteristics that dissipate energy during deformation, which is more critical for applications *in vivo* rather than *in vitro* studies [65]. Moreover, viscoelasticity also influences the smoothness of the printing process and the stability of the printed structure [13]. Therefore, viscoelasticity is an important factor to screen suitable biomaterials for skin substitutes bioprinting.

In addition to complex biomechanical signals, cells are also regulated by biochemical signals from neighboring cells or the surrounding microenvironment [65]. The addition of chemokines and cytokines is an effective means to improve the biochemical properties of bioink. However, the abundance of bioactives in natural skin ECM cannot be mimicked by the addition of one or several factors [43], which leads to more attention is focused on the natural cocktails of microenvironmental factors, such as dECM, exosome and platelet-rich plasma (PRP). Zhao *et al*. constructed a multi-layered skin structure by PRP-containing bioink used for wound treatment. Compared with unmodified bioink, the PRP-containing bioink facilitated vital physiological processes including ECM synthesis, macrophage polarization and angiogenesis [66]. Wang *et al*. reported that a methylcellulose–chitosan hydrogel loaded with placental mesenchymal stem cell (MSC)-derived exosomes can promote skin wound healing in diabetic mice by enhancing vascularization and reducing apoptosis. In addition to neovascularization, hair follicles and glands were observed in the healed region [67].

### Seed cells

As mentioned above, seed cell is important component of bioinks. Skin cells are positioned at high degrees of heterogeneity in a sophisticated microenvironment [68]. To emulate native human skin, the suitable cell type should be determined based on the fundamental structure of skin.

As the largest organ of the human body, skin has developed a three-layered construction [69]. The epidermis can readily regenerate after minor insults, in which keratinocyte is the main cell type [65]. The dermis, mainly comprised of fibroblasts, provides structural support to the epidermis and embedded appendages [69]. The hypodermis, which is mainly consisted of lipocytes, can buffer impact force, store energy and secrete bioactive substances [70]. The presence of appendages (hair follicles, sweat glands, sebaceous glands and nerves, for instance) further increases the structure complexity of skin. Besides keratinocytes and fibroblasts, there are also melanocytes, vascular ECs and immune cells in the skin [69]. Multiple cell types interact with each other and secrete ECM proteins to maintain normal tissue homeostasis and coordinate wound repair [71]. Cellular diversity and spatial structural heterogeneity combine to create the prominent functions of skin such as barrier, thermoregulation and sensation, metabolism, as well as immunity [72].

The basic strategy of skin tissue engineering is to recapitulate epidermis and dermis, which makes fibroblasts and keratinocytes, alone or together, the primary choice [9]. Keratinocytes live across the epidermal region with varying degrees of differentiation [73]. Keratins, a differentiation protein expressed by keratinocytes, help to provide structural supports and to regulate the cell growth and differentiation [74]. Fibroblasts are responsible for the production of ECM, providing skin with sufficient strength and good ductility [75]. Keratinocytes and fibroblasts play an important role in wound healing and pathological development of skin diseases [76]. Though the combination of epidermal keratinocyte and dermal fibroblast is better than being individually used [11], skin equivalents containing only two types of cells simply restore the structure of skin. Great efforts have been made to explore better printing strategies for biomimetic skin models since Lee *et al*. first deposited keratinocytes and fibroblasts in a stratified arrangement using 3D bioprinting in 2009 [77]. Nowadays, more types of functional cells are applied in the research of wound healing, pigmentation, vascularization, appendage regeneration and even the construction of pathological microenvironment in 3D-bioprinted skin.

Melanocytes are given great hope to construct 3D-printed skin with pigmentation. They reside in the stratum basal layer and produce pigment, which helps to protect the skin from ultraviolet rays [78]. Melanocytes are also part of the skin immunological response and display extensive interactions with other immune cells [79]. The use of melanocytes has long been explored in the construction of skin substitutes, but uneven pigmentation is still a great challenge. Recently, Wei *et al*. fabricated a uniformly pigmented human skin model using melanocytes, keratinocytes and fibroblasts. The 3D-bioprinted skin showed resembling morphology and similar pigmentation as the natural skin [21]. It might help to address the psychological and physical problems caused by abnormal pigment after skin substitutes transplantation on patients [78].

Under natural conditions, functional cells in the skin obtain nutrients and oxygen through the adjacent vascular system to maintain physiological functions [46]. When skin is injured, blood vessels transport immune cells and bioactive substances to the wound to regulate the regional microenvironment [69]. Furthermore, the reconstruction of blood vessels using vascular ECs within a skin equivalent can improve the longevity of the model. In practice, by combining ECs with other skin cells, researchers have created perfusable vascularized human skin equivalents [80], vascularized pathological models such as atopic dermatitis model [81] and diabetic skin model [82], and even vascularized skin models that support regeneration of the appendages [83]. It is no exaggeration that the use of ECs to construct vascularized skin models is a great breakthrough in 3D bioprinting technology.

The skin equivalents constructed from the above cell types have shown good results in *in vitro* experiments, but for *in vivo* application, the host immune response must be taken into account [55]. It is difficult to obtain cells from the damaged host autologously, and the excessive cell demand and long culture time limit the clinical application. Therefore, an alternative cell with low immunogenicity and good proliferation or differentiation capacity is needed. Stem cells are the most promising cells to solve these problems [84]. Many types of stem cells have been applied into skin tissue engineering, such as MSCs, adipose‐derived stem cells (ADSCs) and induced pluripotent stem cells (iPSCs). They can regulate the microenvironment and promote wound repairing, vascularization and appendage regeneration through their strong differentiation and secretion abilities [84]. However, it necessary to fully understand the differentiation process when undifferentiated stem cells are used as a source of differentiated cell types. Multiple factors may lead to deviations in therapeutic outcomes, even tumorigenesis [85]. In addition, ethical issues are obstacles that must be faced for stem cell applications [86].

In general, research on different cells for the construction of 3D-bioprinted skin has been successful preliminary, but there is still a lot of work to fully restore the dynamically varying microenvironment. It is a great challenge to precisely regulate the biological behavior of cells so that they can play their proper role without side effects. Furthermore, it is necessary to choose the appropriate cell types flexibly according to the heterogeneity of tissues, the complexity of microenvironment and the differences of individuals.

## Strategies for skin bioprinting

The need for a satisfactory, permanent physiologic replacement of skin as well as a credible and effective model *in vitro* for scientific researches have long been recognized. Initially, skin substitutes mainly consisted of a single type of cell and/or biomaterials with simple structure. Further development mainly focuses on optimizing the process of the skin bioengineering and shortening the lengthy preparation time [9, 87]. Although giant progress has been gained, limitations such as tedious construction steps, lengthy culture time, high cost, low cell viability and vulnerable to infection constrain further scientific studying and clinical application of bioengineered skin [9, 10]. As an advanced, versatile and innovative biofabricating technology, 3D bioprinting provides a promising platform to create highly sophisticated and multicomponent structures to promote skin tissue engineering for wound healing and disease modeling.

Bioprinting strategies for skin constructs mainly include droplet-based bioprinting, extrusion-based bioprinting, digital light processing bioprinting, *in situ* bioprinting, cell spheroid-laden bioprinting, etc. The advantages and disadvantages of these bioprinting strategies are summarized in Table 2.

**Table 2.** Comparison of various strategies for skin bioprinting

Bioprinting strategy	Advantages	Disadvantages
Inkjet bioprinting	Widely used	Thermal and mechanical stress to cells
	High printing speed	Limited printable materials
	High resolution	Low cell concentration
	High cell viability	
	Low cost	
Laser-assisted bioprinting	Non-contacting process	Limited printable materials
	Nozzle free	High cost
	High precision	Time-consuming
	High concentration and high viability of cells	
Electrohydrodynamic jet printing	High precision	High cost
	High structural integrity	Limited printable materials
Extrusion-based bioprinting	Widely used	Limited printing accuracy
	Good compatibility with materials	The need for gelation and shear thinning properties of materials
Digital light processing bioprinting	High printing speed and consistency	The need for photocuring properties of materials
	High structural integrity and mechanical property	
	High precision	
*In situ* bioprinting		
Automated	Automated fabrication process	Complicated scanning modality
	*In situ* cross-linking	Sufficient room for operation
	Minimal invasion	Low degree of freedom
Handheld	Low cost	Experience-dependence
	Portability	Low resolution
	Convenient for sterilization	Non-uniform deposition
Cell spheroid-laden bioprinting	Pre-aggregation of cells	Limited size of spheroids
	Precise positioning and arrangement of cells	Complex steps for construction
4D bioprinting	Recreation of spatiotemporal factors	Limited printable materials
Bioprinted skin-on-a-chip	Presence of biochemical and biomechanical cues	Complex process for construction
	Presence of multi-cell/multi-organ interactions	
Microfluidics-assisted extrusion bioprinting	Precise deposition of hydrogels	Complex process for construction
	Good repeatability	

### Droplet-based bioprinting

Droplet-based bioprinting originates from inkjet printing technology, which could be traced back in the 1950s when the first practical inkjet device was invented [88]. According to the principle of droplet formation, droplet-based bioprinting can be mainly classified into three major categories, namely inkjet bioprinting, laser-assisted bioprinting and electrohydrodynamic jet bioprinting [89, 90].

Inkjet bioprinting can be divided into two categories: continuous-inkjet bioprinting and drop-on-demand inkjet bioprinting. In brief, continuous-inkjet bioprinting relies on the inherent tendency of bioink solution to flow through a nozzle under pressure and subsequently shatter into continuous droplets owing to Rayleigh–Plateau instability. Electrically conductive drops of bioink can be easily steered to their specific locations under the influence of electric or magnetic force. For drop-on-demand inkjet bioprinting, pressure pulses are introduced through a thermal or a piezoelectric or an electrostatic actuator. Continuous-inkjet bioprinters generate droplet at a relatively faster rate, while drop-on-demand inkjet bioprinters generate droplets when required [7, 88]. Inkjet bioprinting has been frequently used in skin engineering and regenerative medicine [9, 88]. Albanna *et al*. creatively combined inkjet bioprinting with wound scanning imaging technology to ensure the accurate delivery of appropriate materials and cells to specific areas of the wound. Results showed that *in situ* inkjet bioprinting of autologous skin cells accelerated wound healing of extensive full-thickness wound [91]. Kim *et al*. modified a 3D bioprinting system by combining inkjet and extrusion printing modules to construct a vascularized skin model where the epidermal compartment was fabricated via inkjet bioprinting. This bioprinted skin model with a functional transwell system revealed favorable biological characteristics including a stabilized fibroblast-stretched dermis and stratified epidermis layers after 14 days, providing a cost-efficient and attractive research platform [33].

Laser-assisted bioprinting relies on laser-induced driving force to propel the cell-laden bioink onto a collector substrate, including laser-guided direct writing and laser-induced forward transfer (LIFT) as well as emerging technologies derived mainly from LIFT (absorbing film-assisted LIFT, biological laser processing, etc.) [89, 92]. This technology is versatile to fabricate tissue constructs with high resolution, high cell density and viability in a nozzle-free and non-contact way. Limitations include high demands on physical properties of bioink, high cost and time-consuming [7, 9]. Koch *et al*. successfully created a simple skin substitute with fibroblasts and keratinocytes embedded in collagen hydrogel via laser-assisted bioprinting. The bioprinted skin substitute precisely mimicked natural skin cell localization and gap junction formation [93].

Electrohydrodynamic jet printing employs an electric field to pull the bioink through the orifice obviating the need for a substantially high pressure. The electric field leads to the accumulation of mobile ions in the bioinks as well as the deformation of suspended bioink meniscus at the orifice. Bioink droplets are ejected when the electrostatic stresses overcome the surface tension at the orifice [88, 90, 94]. Recent studies of electrohydrodynamic printing in the field of skin engineering and regenerative medicine mainly focus on the fabrication of skin scaffolds and novel percutaneous therapy devices such as microneedles and thermotherapy patches [95–98].

### Extrusion-based bioprinting

Extrusion-based bioprinting is the most popular and widely used bioprinting technology to create 3D cell-laden constructs for research and clinical practice [7, 9]. Commercial extrusion-based bioprinter usually includes a bioink dispersing system with one or more printheads, a temperature control module and a program control platform. Extrusion-based bioprinting employs pneumatic pressure or mechanical (usually piston or screw) force to disperse a premixed cell-laden bioink from the nozzle to a collector plate. The printhead together with printing cartridge provides the movement in *x*-, *y*- and *z*-axis. Filaments are generated according to pre-designed 3D CAD models layer-by-layer [9, 89]. Extrusion-based bioprinting demands viscous bioinks so as to maintain the shape of the construct before chemical, thermal or photo-cross-linked [99, 100].

Extrusion-based bioprinting has many advantages such as high cell viability, high compatibility, great flexibility and versatility. Notably, other bioengineering technologies, e.g. coaxial bioprinting, multi-material bioprinting and microfabrication technologies, can be combined with extrusion-based bioprinting for skin bioengineering [33, 82, 101, 102]. Micro-extrusion bioprinting has boosted models with great potential to emulate the microstructure of natural skin [103]. The pioneering work of Huang *et al*. demonstrated successful regeneration of sweat glands in the extrusion-bioprinted niche [24, 104]. Other applications of extrusion-based bioprinting, including cutaneous wound healing, vascularized substitutes formation, skin appendages regeneration and skin disease modeling were elaborated by recent fantastic reviews [58, 105, 106].

### Digital light processing bioprinting

Digital light processing bioprinting, as a kind of photocuring-based bioprinting, utilizes photosensitive polymers and precisely controlled lighting to obtain constructs with high resolution and stable structure [89]. Recent researches on photoactivated materials and bioprinting strategies have been reviewed by Lee *et al*. In brief, exciting progress has been made in the field of photocuring materials, especially in the construction of cell-laden scaffolds [107]. Digital light processing bioprinting has a considerable advantage in printing speed and consistency, and improves structural integrity and mechanical property [9]. Zhou *et al*. employed digital light processing bioprinting to fabricate a functional skin substitute with integrated vasculature. The well-formed interconnected microchannels facilitated gas/nutrient exchange which supported better cell behaviors *in vitro*, and the deposition of well-organized collagen fibers as well as the regeneration of skin appendages indicated the faster and better healing process *in vivo* [83].

### *In situ* bioprinting

Significant progress has been made in developing 3D bioprinting technologies and versatile bioinks to facilitate complex tissue regeneration. However, most of the traditional bioprinting strategies form bioprinted constructs with specific shapes on flat surfaces, resulting in the unmatched bioprinted constructs to the defect site. Besides, bioprinted constructs in petri dish do not adhere properly to the host tissue [108, 109]. *In situ* bioprinting, also known as *in vivo* bioprinting or intraoperative bioprinting, literally directs depositing bioinks inside the defect *in vivo* and has been introduced as an alternative strategy for translation of bioprinting from bench to bedside [110, 111]. Samandari *et al*. systemically reviewed the emerging *in situ* bioprinting strategies of various kinds and specific requirements of bioinks for *in situ* bioprinting [108]. With an *in situ* bioprinter, the operator can immediately apply the treatment by manufacturing customized constructs which accurately match the defect geometry and *in situ* cross-linked to enhance the mechanical strength. Skin was identified as an optimal target organ to perform *in situ* bioprinting without open surgery [111]. *In situ* bioprinting can be classified into two major categories, namely automated *in situ* bioprinting and handheld *in situ* bioprinting.

Automated *in situ* bioprinting is similar to most conventional bioprinting strategies in terms of the automated mode. Once the computer-aided design is loaded into the software, the bioprinter automatically fabricates a 3D scaffold. The automated system ensures high printing accuracy, rapid fabrication rate, minimal invasion and human errors [108, 110, 112, 113]. The drawbacks are the complicated scanning modality, more room for operation and low degree of freedom. Modulations such as adaptive bioprinting and error compensation before bioprinting have been adopted to solve these problems. Various bioprinting strategies, such as extrusion-based bioprinting, inkjet bioprinting and stereolithography bioprinting, have been adapted for automated *in situ* printing for the treatment of skin wounds (Table 3).

**Table 3.** Application of *in situ* bioprinting in skin regeneration and wound repair

Printing strategy	Scanning approach	Bioink	Laden cells	Models	Ref.
Automatic					
Extrusion-based bioprinting	NA	Fibrinogen + collagen	AFSCs, BMSCs	Murine full-thickness skin wound model	[118]
	NA	Thiolated HA + thiolated gelatin + PEGDA	AFSCs	Murine full-thickness skin wound model	[119]
(For soft tissue repair) droplet-based bioprinting	3D laser scanner	(For soft tissue repair) fibrinogen + collagen + KGF	(For soft tissue repair) dermal fibroblasts	Murine composite skin and calvarial bone defect	[114]
Inkjet-based bioprinting	3D laser scanner	Fibrinogen + collagen	Dermal fibroblasts, epidermal keratinocytes	Murine and porcine full-thickness skin wounds	[91]
Two-photon bioprinting	NA	HCC-PEG	Muscular fibroblasts	Printing inside murine normal dermis	[111]
Handheld					
Extrusion-based bioprinting	Not required	GelMA + VEGF	NA	Porcine full-thickness skin wound model	[109]
		Fibrinogen + HA	UMSCs	Porcine full-thickness skin burn injury model	[116]
		Alginate + collagen; fibrinogen + HA + collagen	(For *in vitro* experiments) dermal fibroblasts, epidermal keratinocytes	Murine and porcine full-thickness skin wounds	[117]
		Alginate + gelatin + PRP	Dermal fibroblasts, epidermal stem cells	Murine full-thickness skin wound model	[66]

AFSCs, amniotic fluid-derived stem cells; BMSCs, bone marrow-derived mesenchymal stem cells; GelMA, gelatin methacryloyl; HA, hyaluronic acid; HCC, 7-hydroxycoumarin-3-carboxylate; KGF, keratinocyte growth factor; NA, not applicable; PEG, polyethylene glycol; PRP, platelet-rich plasma; UMSCs, umbilical cord-derived mesenchymal stromal cells; VEGF, vascular endothelial growth factor.

Handheld *in situ* bioprinting is recognized as a simplified and promising bioprinting strategy, which is more economical, portable, minimal-invasive and easy to be sterilized [108]. Several handheld *in situ* printers have been developed to treat skin wounds, most of which rely on extrusion-based bioprinting (Table 3). As a manually operated and experience-dependent technology, handheld *in situ* printing has its inherent weaknesses such as lower resolution, non-uniform deposition and lack of precision in reconstruction of anatomically correct shapes [108, 114]. Alternative strategies, e.g. employing a human-controlled robotic-assisted bioprinting system, have been developed as remedies for handheld *in situ* bioprinting [115–117].

### Cell spheroid-laden bioprinting

**Figure 2.**
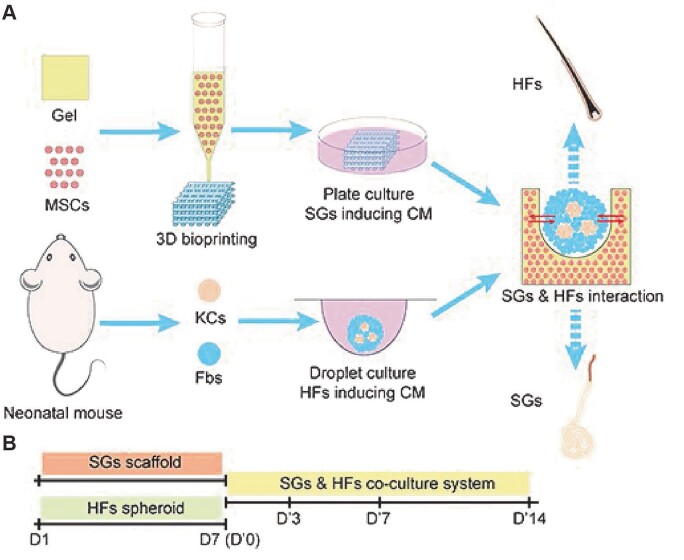
Establishment of 3D skin constructs with multiple appendages. (**A**) Schematic diagram showing the procedure to establish 3D skin constructs *in vitro*. (**B**) Time points used in inducing SGs and HFs separately and SG–HF co-culture. Adapted with permission from Zhang *et al*. [125].

Owing to the distinct characteristics including self-renewal ability and high proliferative potential for different lineage differentiation, a variety of stem cells have been applied to bioprint 3D skin substitutes [103]. The precise positioning and arrangement of stem cells are essential for the reconstruction of complex tissue architecture [120, 121]. Cell spheroids and aggregations have advantages to mimic the native structure and microenvironment [120, 122, 123]. Combining cell spheroids with 3D bioprinting technologies, cell spheroid-laden bioprinting has increasingly been employed to answer a wide variety of clinical and biomedical inquiries ranging from pathophysiological and pharmacology research to tissue engineering and regeneration [102, 122–124]. Recent progress of cell spheroid-laden bioprinting in skin engineering mainly focuses on appendages regeneration. For example, a novel sweat gland scaffold was fabricated via extrusion-based bioprinting with hair follicle spheroids seeded on top, realizing the symbiosis of sweat glands and hair follicles *in vitro* (Fig. 2) [125].

### Other strategies

In addition to the strategies reviewed above, other emerging technologies, for instance, four-dimensional (4D) bioprinting [126–128], bioprinted skin-on-a-chip [129–131], and microfluidics-assisted extrusion bioprinting [9, 132–134], are being or to be applied for skin tissue engineering and wound healing researches as well. Derived from 3D bioprinting, 4D bioprinting may have the potential to recreate the spatiotemporal changes in tissue geometry and transformations in the spatial distribution of cells and ECM [128]. Recently, Douillet *et al*. developed a versatile *in vitro* tool by means of patterning fibroblasts on collagen gels via laser-based 4D bioprinting to investigate different aspects of matrix remodeling. This model contributed to a better understanding of morphogenetic mechanisms, dermal organizations and remodeling process in healing [126]. In terms of wound healing, pH and temperature-responsive bioprinted multifunctional dressings were fabricated to provide signals of wound inflammation as well as release the drugs as needed [135, 136]. Skin-on-a-chip technology combines advancements in tissue engineering and microfluidics to reproduce critical functions of natural skin. The key features of the skin-on-a-chip include the presence of biochemical and biomechanical cues and the integration of multiple cell types to model the intricate interactions. Mori *et al*. fabricated ECs-coated vascular channels within a cultured skin equivalent to form a skin-on-a-chip. The skin-on-a-chip, which contained vascular channels with good delivery and absorption functions, proved to be a promising platform for drug development, cosmetics testing and skin biology research [130]. Microfluidics-assisted extrusion bioprinting has been explored for the precise fabrication of skin constructs in recent decades. Leng *et al*. developed a 3D bioprinting system based on a microfluidics approach to print human fibroblasts-laden alginate hydrogel into a sheet-like format to fabricate skin tissue-like structures. Further results indicated a faster healing and keratinization in a murine wound model [137]. Cheng *et al*. developed a handheld bioprinter with a microfluidic printhead for uniform distribution of MSC-containing fibrin gel directly to the wound, which promoted re-epithelialization, dermal cell proliferation and neovascularization in a porcine full-thickness burn model [116].

## Applications of 3D skin bioprinting

### 3D-bioprinted skin constructs for wound healing

Wound healing process is a coordinated cascade of interacted events to restore the skin integrity, involving an integrated action of multiple cells and bioactive molecules [9, 11, 138]. Typically, wound healing process can be sectioned into four overlapping and consistent phases, namely hemostasis, inflammation, proliferation and remodeling. After injury, platelets and the clotting cascade are quickly activated, releasing cytokines, inflammatory and growth factors. Fibrin along with platelets, red blood cells and inflammatory cells forms a temporary protective layer at the wound site [87]. Neutrophils and monocyte–macrophages as well as various growth factors and cytokines take part in the removal of debris and foreign matter [139]. The tissue repair process starts with the formation of granulation tissue and is intensified by the migration and proliferation of fibroblasts, keratinocytes and ECs, namely collagen deposition, epithelialization and angiogenesis. At the final stage of wound healing, collagen is rearranged to form regularly oriented collagen bundles under the effect of mechanical force and other factors. In the meanwhile, the fibroblast differentiates into the myofibroblast phenotype, which is considered a characteristic feature of scarring [140, 141].

Skin wound is common in daily life. However, injuries to large surface areas, such as massive burns and extensive trauma, as well as refractory wounds with underlying diseases complicate the healing process. 3D-bioprinted skin constructs are considered to be ideal models for the research of skin cell behavior and wound repair process under different conditions. Douillet *et al*. fabricated an *in vitro* model that could replicate fibroblast–myofibroblast dynamic remodeling. Fibroblasts were patterned on collagen gels with laser-assisted bioprinting and the results showed that the maturation of anisotropic fibroblast patterns, instead of myofibroblasts, resulted in collagen anisotropic reorganization [126]. Sarmin *et al*. employed dECM-enhanced multicellular (immortalized keratinocytes, dermal fibroblasts and THP-1 monocytes) fibrin bioink to develop a flexible and tunable model for wound healing. The bioink was deposited on a sectionalized silicone frame and Pluronic F127 was employed as a sacrificial wound template to generate wounds of varying sizes and shapes [142]. Kim *et al*. fabricated a diabetic skin model with structural similarities and diabetic properties by combining inkjet, extrusion and coaxial bioprinting with a PCL transwell system. Human epidermal keratinocytes, diabetic dermal fibroblasts, diabetic preadipocytes and human umbilical vein endothelial cells (HUVECs) were encapsulated in different bioinks respectively. To create a wound, a printing needle was located at the center of the model. Typical hallmarks in diabetes, such as insulin resistance, adipocyte hypertrophy, inflammatory reactions, vascular dysfunction and slow re-epithelization after wound, were successfully observed in the diabetic skin model [82].

The restoration of skin structure and function remains an intractable problem to date. 3D bioprinting offers an attractive and competitive solution to construct patient-specific skin grafts with multi-layered biomimetic structures [143]. Jin *et al*. utilized acellular dermal matrix-enhanced gelatin methacryloyl (GelMA) bioink to develop a novel 3D full-thickness skin construct where human epidermal (HaCaT) cells, fibroblasts and HUVECs were, respectively, encapsulated into different concentrations of GelMA to simulate the stratified structure of the natural skin. The bioprinted functional skin accelerated wound healing and re-epithelization, and stimulated dermal ECM secretion and angiogenesis [144]. Zhao *et al*. employed PRP-integrated alginate–gelatin bioink to fabricate 3D skin constructs embedded with dermal fibroblasts and epidermal stem cells via *in situ* extrusion bioprinting. The skin constructs accelerated wound closure, modulated the inflammation process and initiated angiogenesis at the wound site [145]. Compared to conventional skin tissue engineering approaches, 3D bioprinting offers many advantages in complex structure construction, spatial integration and reproducibility. In addition, bioprinted skin constructs may help to improve regeneration and decrease intervention, thus conducive to obtain better clinical outcomes [9, 87].

### 3D skin bioprinting for appendages regeneration

Skin appendages, including sweat glands, hair follicles and sebaceous glands, are responsible for maintaining skin barrier function, regulating body temperature and aesthetic function [146]. Abundant researches revealed the origination and regulatory mechanism of skin appendages with their microenvironment [147–149]. *In vitro*-induced differentiation of skin appendages and symbiotic culture of them have been achieved in labs, and multifarious bioengineered skin equivalents seeded with induced stem cells or spheroids have been constructed, which promoted the functional regeneration of skin tissues at the wound site [24, 125, 146, 150]. Notably, recent studies have reported remarkable results of the reconstruction of skin with functional appendages via 3D bioprinting [24, 151, 152].

#### Sweat gland

**Figure 3.**
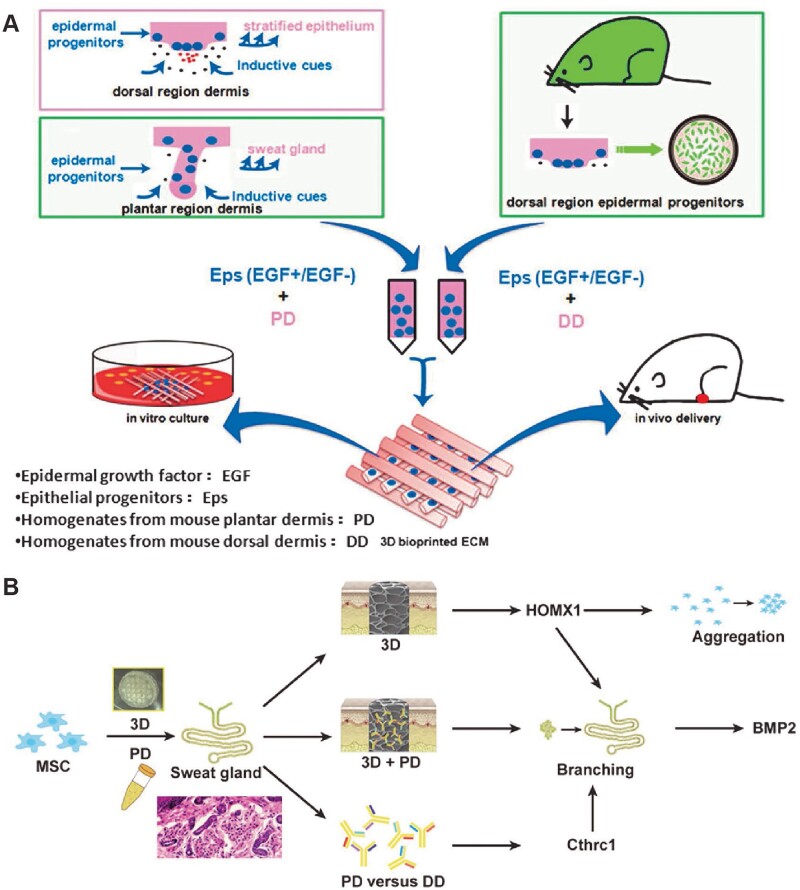
*In vitro* bioprinted microenvironment for sweat gland regeneration. (**A**) Schematic of the printing process with epidermal progenitors and ECM incorporated in composite hydrogels to mimic the microenvironment for sweat gland regeneration. (**B**) The graphic illustration of 3D-bioprinted matrix directed MSC differentiation. CTHRC1 is the main biochemical cues during SG development, and structural cues up-regulated the expression of *Hmox1* synergistically initiated branching morphogenesis of SG. Adapted with permission from Huang *et al*. and Yao *et al*. [24, 153].

Sweat glands can be divided into eccrine sweat glands and apocrine sweat glands, of which the former is mainly responsible for thermoregulation and sweating. Patients with extensive trauma and deep burn would suffer from sweat gland regeneration disorder and endure the pain of being unable to sweat and dissipate heat in hot environment. In terms of *in vitro* reconstruction of the sweat glands, Huang *et al*. initiatively fabricated 3D-bioprinted ECM containing mouse PD homogenate to create an inductive niche for sweat gland differentiation of mouse epidermal progenitor cells. Meanwhile, *in vivo* transplantation of the bioprinted constructs resulted in functional restoration of sweat glands at the burned paws of mice (Fig. 3A) [24, 104]. Yao *et al*. employed extrusion-based bioprinting to construct a sweat gland microenvironment *in vitro* to drive MSCs differentiation into functional sweat gland. Biochemical and structural cues were identified as two critical impacts of 3D-printed matrix on MSCs fate decision into the glandular lineage. In addition, the results revealed that Collagen Triple Helix Repeat Containing 1 (CTHRC1), a critical biochemical regulator for sweat gland specification, and heme oxygenase 1 (*Hmox1*), an important gene involved in MSCs differentiation and biomechanical activation, were two essential factors to boost sweat gland gene expression profile (Fig. 3B) [153]. Song *et al*. comprehensively reviewed recent progress in bioprinting-assisted sweat gland reconstruction and innovatively outlined different aspects of the biomimetic microenvironment indispensable for sweat gland regeneration [106].

#### Hair follicle

Hair follicles are composed of hair papillae, hair matrix, root sheath and hair bulges. *In vitro* reconstruction and *in vivo* regeneration of hair follicles have always been a hot issue. Recent studies revealed a road map for the development of hair follicles, providing guidance for the construction of hair follicles. In terms of bioprinting for hair follicles, Zhang *et al*. reported the symbiosis of sweat glands and hair follicles *in vitro*: 3D-bioprinted porous construct containing induced sweat gland cells was seeded with hanging drop cultured hair follicle spheroids to permit interdependent regeneration of sweat glands and hair follicles. The mutual inhibitory effect was revealed in this 3D-bioprinted skin constructs, indicating the sequential genesis of multiple appendages in skin regeneration (Fig. 2) [125]. Kang *et al*. fabricated a multi-layered scaffold for dermal papilla cells to regenerate entire hair follicles *in vivo* via 3D bioprinting. Fibroblasts, HUVECs, dermal papilla cells and epidermal cells were encapsulated in gelatin–alginate hydrogel, respectively, to bioprint each layer of the scaffold to simulate the microenvironment required for hair follicle reconstruction [152]. Nanmo *et al*. proposed an efficient approach for the scalable and automated preparation of highly hair-inductive grafts by multiple nozzle bioprinting [151]. Lian *et al*. employed coaxial vertical bioprinting to reconstruct human hair follicles in a multi-segment filament manner. Human dermal papilla cell-laden GelMA and human keratinocyte-laden gelatin were simultaneously but independently embedded in human dermal fibroblast-laden GelMA, thus providing complex microenvironments for the regeneration of hair follicles [101].

#### Sebaceous gland

Sebaceous glands are composed of numerous acini branching off a central duct [154]. The regeneration of sebaceous glands has also attracted attention in recent years. In terms of *in vitro* reconstruction of the sebaceous glands, Chen *et al*. employed poly-caprolactone, which was coated with decellularized matrix of ADSCs, to fabricate tarsal plate scaffolds via 3D printing technology. The biocompatible scaffolds were seeded with immortalized human SZ95 sebocytes. *In vivo* experiments revealed excellent sebocytes proliferation and lipogenesis on the scaffolds, which made it a feasible strategy for tarsal plate regeneration [155].

### 3D skin-bioprinted models for *in vitro* medical searches

Animal models and two-dimensional (2D) cultured cells are not always effective at discovering human pathophysiological and toxicological responses. Numerous instances of significant differences between the disease phenotypes observed in animal models and humans have been reported [76, 156, 157]. Cells in 2D petri dish could not exactly mimic native cell behavior and the complexity interactions with the microenvironment *in vivo* [158]. In comparison, *in vitro* physiological and pathological 3D skin models can overcome these limitations and replicate *in vivo* biomechanical and biochemical cues, providing an efficacious and promising platform for mechanism/therapeutic research and pharmaceutical/cosmetic screening.

**Figure 4.**
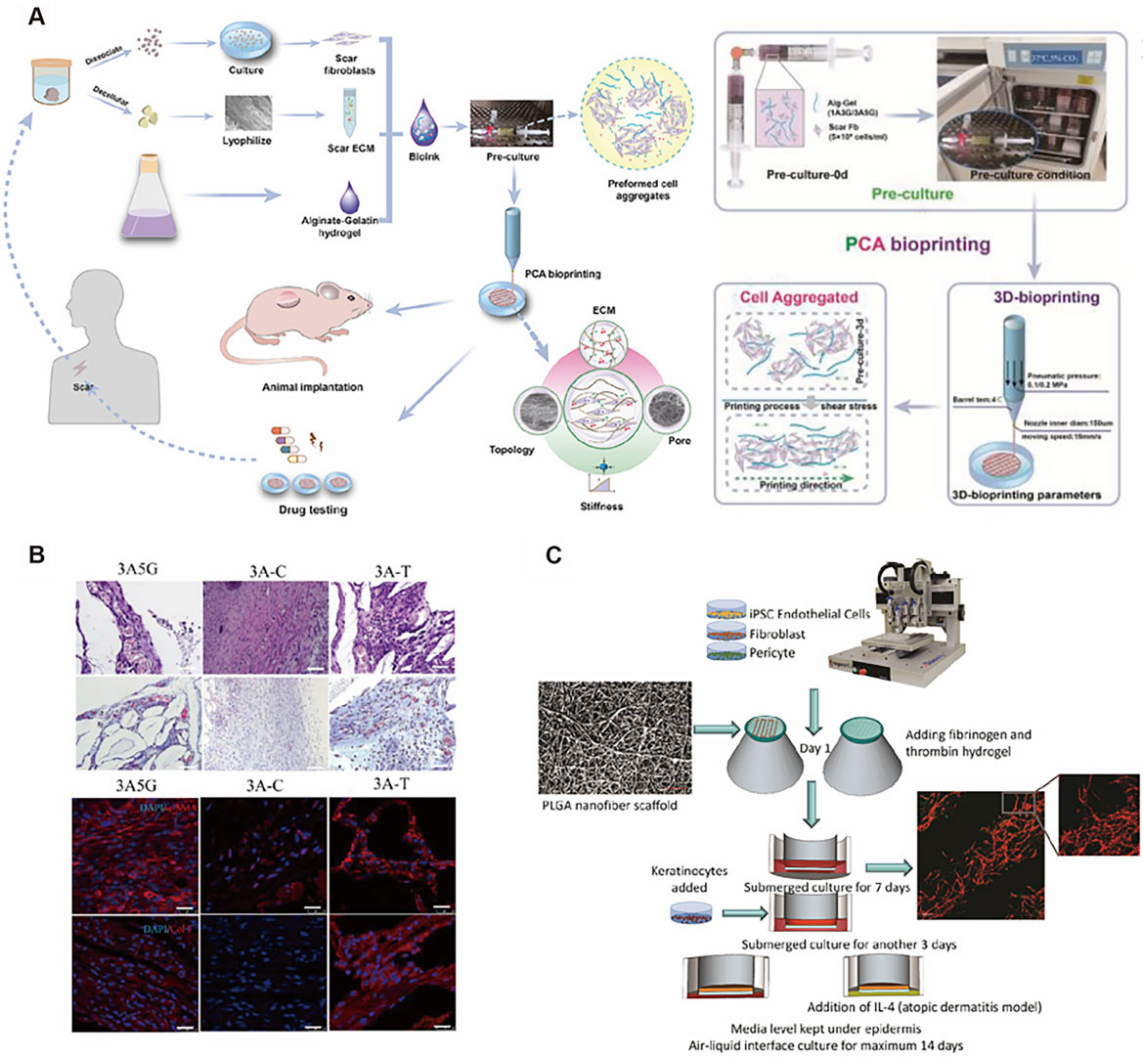
Modeling human hypertrophic scars and atopic dermatitis with 3Dbioprinting. (**A**) Schematic illustration of the whole process of fabrication of scar model and the details of PCA bioprinting. (**B**) HE (scale bar = 200 μm), Masson images (scale bar = 200 μm) and the expression of myofibroblasts markers of scar tissue in different groups (α-SMA, col I: red, DAPI: blue, scale bar = 100 μm). 3A5G: 3A5G+SFb+scar ECM, 3A–C: 3ASS+ cobimetinib, 3A–T: 3ASS+ triamcinolone acetonide. (**C**) Bioprinting steps of vascularized full-thickness skin equivalent and diseased condition. Poly(lactic-co-glycolic acid) nanofibers scaffold was taken using Keyence 3D laser scanning confocal microscope (scale bar: 20 μm). Vascularization in the dermis equivalent after 1 week of culture (CD31, red, laminin, green, scale bar: 200 μm). Adapted with permission from Liu *et al*. [81] and Bin *et al*. [159].

As an emerging fabrication method allowing automated, standardized, massive, and faster production, 3D bioprinting is employed to create multifarious skin disease models, such as hypertrophic scar models, atopic dermatitis models and melanoma models. Yao *et al*. fabricated scar dECM-enhanced alginate–gelatin hydrogel to construct a 3D functional human hypertrophic scar model by preformed cellular aggregates bioprinting (Fig. 4A). Gene and protein expression showed that this model exhibited characteristics of early-stage hypertrophic scar. Responsiveness to clinical anti-scarring drugs demonstrated its potential for drug testing (Fig. 4B) [159]. Liu *et al*. employed human keratinocytes, fibroblasts, pericytes and iPSC-derived ECs to fabricate a vascularized full-thickness skin equivalent via 3D bioprinting, in which interleukin-4 was added to generate the atopic dermatitis model. Specific clinical hallmarks present in the models, such as spongiosis and hyperplasia, differential expression of differentiation proteins and increased levels of pro-inflammatory cytokines, demonstrated its successful construction. Anti-inflammatory corticosteroid and Janus Kinase inhibitors for atopic dermatitis resulted in morphology and functional restoration in this bioprinted models (Fig. 4C) [81]. Duan *et al*. used 3D bioprinting to construct GelMA-polyethylene (glycol) diacrylate composite scaffolds for human malignant melanoma cell growth. The results showed a high aggregation, expansion and differentiation of melanoma cells on the bioprinted scaffolds [160]. Similarly, Jeong *et al*. constructed a bioprinted collagen scaffolds which could improve the maintenance and survival rate of cryopreserved patients-derived melanoma explants, providing an alternative way to *in vitro* construct melanoma models [161].

3D-bioprinted skin models and skin-on-a-chip models enable the pharmaceutical or cosmetic screening in a high-throughput, high-fidelity, cost-efficient and time-saving manner. Both pharmaceuticals and cosmetics must be assessed for potential toxic and allergic effects prior to the further testing such as pharmacokinetic and pharmacodynamic studies [162–164]. Especially, with the total ban of animal trials for cosmetic purposes in growing numbers of countries and regions, there is a strong demand for skin substitutes that could serve as an alternative to animal experiments [164]. Various engineered skin models have been developed to meet the urgent need [132, 163]. Some skin-on-a-chip models combine the basic components with perfusion pumps or even with other organ-on-a-chip models or organoids to examine multi-cell and multi-organ interactions especially in more complicated cases [129, 134]. Although most of them remain to be further validated in terms of both the fabrication process and the efficacy in toxicity and allergenicity testing, they offer the possibility of measuring the impact of subjects on skin main compartments with high throughput and efficiency.

## Vascularization of 3D-bioprinted skin substitutes

**Figure 5.**
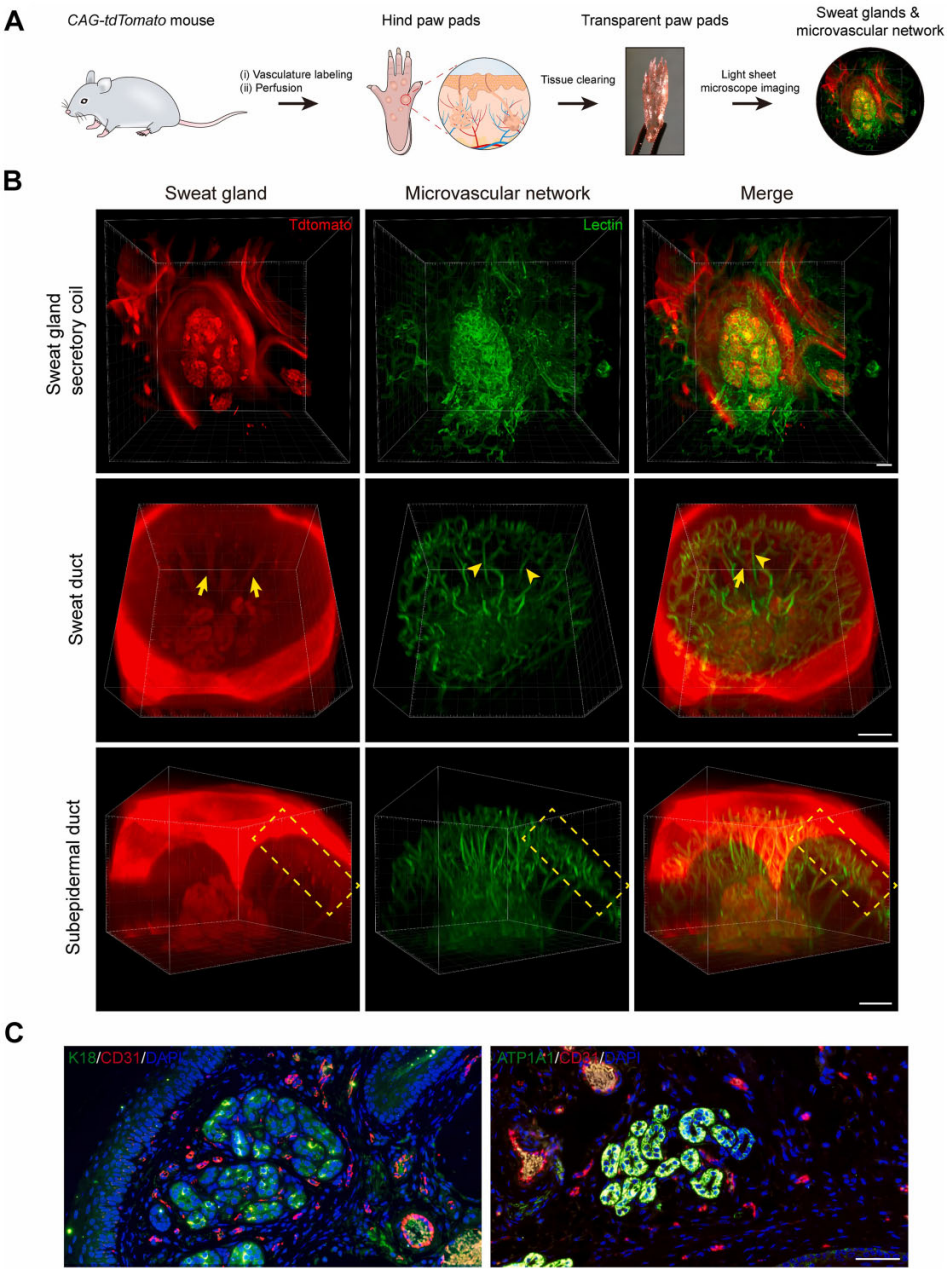
Determine the anatomy of SGs and their surrounding microvasculature using tissue clearing technique and histological staining. (**A**) Schematic showing 3D imaging of the SG-vasculature anatomical structure by using the tissue-clearing technique. (**B**) Light-sheet microscopy images showing the close proximity of SGs and their vascular beds after optical clearing. Arrows and arrowheads indicate SGs and microvessels, respectively. Dashed boxes highlight the subepidermal ducts and nearby vascular arcades. (**C**) Conventional histological sections confirming the capillaries (CD31) closely surround the SGs (K18 and ATP1A1). CD31, red; K18, ATP1A1, green; DAPI, blue. Scale bars: (B) 100 μm; (C) 50 μm. Adapted with permission from Yuan *et al*. [167].

The complex and mature vasculature of the skin structure creates a dynamic microenvironment, which is critical to the function of skin tissue in both physiological and pathological conditions [165]. The role of blood vessels in the skin is not limited to the transport of nutrients; in the case of hair follicles, the interactions with blood vessels regulate the activation of hair follicle stem cells to maintain homeostasis, which may have positive implications for hair follicle regeneration [166]. Recently, Huang *et al*. further found that blood vessels are inextricably associated with the morphogenesis and regeneration of sweat glands via tissue clearing technique (Fig. 5) [167], which suggested that vascularization may be an indispensable step in the construction of skin substitutes containing functional appendages. Moreover, dynamic development of the vascular system is present at every stage of the wound healing process, and abnormalities at any stage can lead to abnormal healing [69, 168]. Changes of the vascular system also contribute to the development of many skin diseases, especially autoimmune diseases and skin tumors [169, 170]. Therefore, researches in skin bioprinting have increasingly focused on the realization of vascularization to realistically reproduce the skin microenvironment in recent years.

Many methods have been explored to construct vascularized 3D printed skin, which can be roughly divided into pre-vascularization *in vitro* and induced vascularization *in vivo* [171]. The most common method for pre-vascularization *in vitro* is performed by using sacrificial materials. In other words, sacrificial biomaterials are first embedded in the printed structure, followed by dissolving the sacrificial biomaterials to form hollow channels, and finally perfusing vascular ECs, which can adhere to the channel surface and form vascular-like structures [172]. More recently, coaxial bioprinting, one technique of extrusion-based bioprinting for stereoscopic concentric structure, makes large-scale fabrication of vascularized tissue a reality without complex bioink removal procedures [173, 174]. Hong *et al*. described a vascularized construct by using gelatin–PEG–tyramine prepolymer containing fibroblasts as a shell layer encircling gelatin-based bioink containing ECs via coaxial bioprinting. After *in vitro* incubation, the construct formed an ECs-lined hollow structure and maintained for up to 8 days *in vitro*, which provided new ideas for bioprinting of perfusable multicellular blood vessel in skin substitutes [175].

However, blood vessels formed directly by bioprinting *in vitro* relatively deviate from the complex network of microvessels in the skin in terms of diameter and morphology [165, 176]. Inducing formation of blood vessels *in vivo* by adding growth factors or modulating mechanical and topological cues of the printed tissue after transplanted is expected to overcome this challenge. Vascular endothelial growth factor [177], basic fibroblast growth factor [178], platelet-derived growth factor [179] and stem cell-derived exosomes [180] have been proved to promote neovascularization effectively. Meanwhile, ECs can sense the mechanical signals and microtopography of the 3D environment and change their behavior [181]. Stiffer matrix inhibits migration of ECs and microvascular assembly [182, 183]. Another research showed that uniaxially aligned scaffolds can improve pre-vascularization using porous freeze-dried collagen scaffolds compared with randomly oriented pores [184].

The self-assembly ability of ECs also deserves to be taken into account. Baltazar *et al*. described an implantable multi-layered vascularized bioengineered skin graft using 3D bioprinting. The 3D printed construct is composed of human keratinocytes, fibroblasts, ECs and placental pericytes. *In vitro*, the placental pericytes and ECs self-assemble into interconnected microvascular networks. After implanted, the networks inosculated with mouse microvessels within the wound bed and become perfused in 4 weeks [185]. What cannot be ignored is that inducing neovascularization *in vivo* is less controllable than constructing vascular channels *in vitro* directly before transplanted, potentially leading to excessive vascularization and causing side effects.

All of the above developments have contributed to the advancement of vascularized printed skin, but there are still many issues that need to be addressed. The vascular system within the skin is composed of a network that span multiple orders of magnitude in diameter [186]. It contains arterial, venous and microcirculatory systems with different structure, but most of the current methods for pre-vascularization *in vitro* only construct a tubular single-cell layer [187]. Recapitulating the complex multiscale vascular architecture is still a great challenge. In addition to being a transport channel, blood vessels should also act as a tight barrier, which is necessary to be evaluated after constructed [181]. The issue of the source of ECs deserves equal attention because of the limited amount of autologous supply and the low efficiency of induction using stem cells. Finally, the construction of skin disease models containing specific vascular alterations remains a major obstacle. There is still a long way for the exploration of vascularized skin models.

## Limitations

Numerous 3D skin bioprinting strategies have been reported in the recent decades, which could be regarded as a giant leap for skin tissue engineering. However, few of them have been applied for clinical translation. It is still a long way to finally achieve the functional regeneration of skin by 3D bioprinting. There are several limitations of current 3D skin bioprinting research and development. First, the biocompatibility and mechanical strength of bioink is compromised by the pursuit of perfect printability. For example, collagen owns perfect skin compatibility but poor extrusion property and mechanical strength, which should be modified by mixing printable biomaterials with less compatibility, like gelatin and sodium alginate [188]. Therefore, it is still a challenge to discover perfect biomaterial or blend biomaterials with both promising printability and compatibility for 3D skin bioprinting. Second, there are no efficient vascularization strategies for bioprinted skin substitutes. Vascularization is the first to determine the survival of bioprinted skin substitutes on recipient wound bed. To date, existing vascularization strategies for 3D skin bioprinting, however, could not achieve vascular anastomosis between skin substitute and recipient wound bed within 72 h, indicates lower survival rate of bioprinted skin than skin grafts [189]. Finally, 3D-bioprinted full-functional skin substitute with nerve, vessel and lymph integration is still a theoretical model in lab. The full-function relies on the coordination of bioprinted skin appendages with recipient nerve, vessel and lymph [190]. For example, bioprinted sweat glands embedded in skin substitutes could be functionalized by recipient neural control and sufficient blood supply for exocrine sweat [191]. It is the main difficulty to figure out the interaction between skin appendages and nerve/vessel in future research.

Additionally, the problems in clinical translation of 3D skin bioprinting, such as ethical issues of cell therapy, large-scale production, preservation and transportation, should be taken into consideration during the development of 3D-bioprinted skin substitutes. The solutions to above problems will contribute to the marketing of 3D skin bioprinting in the future decades.

## Future perspectives

The need for a satisfactory, permanent physiologic replacement of skin as well as a credible and effective model *in vitro* for scientific researches have long been recognized. Significant advances in wound repair have been made since 1980s with various skin substitutes being developed for extensive burn injury [7, 9, 192, 193]. Although giant progress has been gained, limitations constrain further clinical translation of bioprinted skin, such as compromised biocompatibility, low mechanical strength, insufficient vascularization and dysfunction.

As the development of material science and advanced manufacturing industry, further 3D skin bioprinting research will mainly focus on optimizing function and developing standard system. Biomimetic bioinks would be discovered to mimic structural and functional heterogeneity of native skin [99, 100]. Efforts such as combining with growth factors delivery and stem cell therapy will achieve better functional outcomes [194–197]. Integration of functional modules to 3D-bioprinted skin should be feasible and customized. Quality standard system of bioprinted skin substitutes and clinical pathway of would treatment with 3D skin bioprinting should be established and completed. Finally, 3D skin bioprinting will be the pioneer to bridges the gap between 3D bioprinting and clinical translation.
